# Emergence of Feline Sporotrichosis near Brazil Border, Argentina, 2023–2024

**DOI:** 10.3201/eid3105.241882

**Published:** 2025-05

**Authors:** Katherina Alicia Vizcaychipi, María Cecilia López-Joffre, Mónica Martínez, Jerson Andrés Cuéllar-Sáenz, Marina Ramos, Celeste Agüero, Natalia Olsina, Emanuel Grassi, Esteban Couto, Jorge Mendoza, Carolina Melchior do Prado, Jorge Pablo Castillo, Mabel D. Giménez, Karen E. DeMatteo, Álvaro A. Faccini-Martínez, Mariana Viale, Adriana Toranzo, Cristina Elena Canteros

**Affiliations:** Instituto Misionero de Biodiversidad, Puerto Iguazú, Argentina (K.A. Vizcaychipi, M. Martínez, E. Grassi); Universidad del Salvador, Virasoro, Argentina (K.A. Vizcaychipi, J. Mendoza, J.P. Castillo); Instituto Nacional de Medicina Tropical Administración Nacional de Laboratorios e Institutos de Salud–“Carlos G. Malbrán,” Puerto Iguazú (K.A. Vizcaychipi, E. Couto); Instituto Nacional de Enfermedades Infecciosas, Administración Nacional de Laboratorios e Institutos de Salud–“Carlos G. Malbrán,” Buenos Aires, Argentina (M.C. López-Joffré, M. Viale, A. Toranzo, C.E. Canteros); Universidad Nacional de Colombia, Bogota, Colombia (J.A. Cuéllar-Sáenz); Dirección Municipal de Zoonosis, Puerto Iguazú (M. Ramos, C. Agüero); Clínica Veterinaria, Puerto Iguazú (N. Olsina); Universidad Federal de Paraná, _Curitiba_, Brazil (C. Melchior do Prado); Instituto de Genética Humana de Misiones, Consejo Nacional de Investigaciones Científicas y Técnicas, Posadas, Argentina (M.D. Giménez); Universidad Nacional de Misiones, Posadas (M.D. Giménez); Washington University in St. Louis, St. Louis, Missouri, USA (K.E. DeMatteo), WildCare Institute at the Saint Louis Zoo, St. Louis (K.E. DeMatteo); Hospital Militar Central, Bogota (Á.A. Faccini-Martínez); Universidad Militar Nueva Granada, Bogota (Á.A. Faccini-Martínez)

**Keywords:** sporotrichosis, zoonoses, fungi, communicable diseases, emerging, One Health, cats, Argentina

## Abstract

We describe a large urban outbreak of feline sporotrichosis caused by *Sporothrix brasiliensis* fungi in Argentina. Over a 7-month period in Puerto Iguazú, which borders Brazil, we identified culture-proven sporotrichosis in 9 cases across 7 households. Public health officials should coordinate cross-border One Health actions and institute context-specific interventions.

Sporotrichosis is an implantation mycosis caused by thermal-dimorphic fungi belonging to the *Sporothrix schenckii* complex (*1*). Among pathogenic species, *S. brasiliensis* has high virulence, epidemic potential, and zoonotic/enzootic transmission that occurs through bites, scratches, or contact with exudates from infected animals, particularly domestic cats (*2*–*4*).

In South America, *S. brasiliensis* was first identified in Brazil and has since been reported in other Latin America countries (*1*–*8*). Over recent decades, sporotrichosis in Brazil has seen a substantial epidemiologic shift, marked by intense, widespread urban zoonotic outbreaks, initially concentrated in Rio de Janeiro, affecting cats, dogs, and humans (*2*,*9*). Those outbreaks have spread to several cities in the southern and southeastern states, including Foz do Iguaçu, located on the Triple Frontier (Argentina, Brazil, and Parguay) between Argentina and Paraguay (*2*,*3*,*5*,*7*,*10*). Recently, a cat (*Felis catus*) infection by *S. brasiliensis* was reported in Ciudad del Este in Paraguay (*8*). In Argentina, the first human isolation of *S. brasiliensis* was documented in 1986 in the south of province of Misiones with no identified source of infection (*4*). Since then, zoonotic sporotrichosis cases have increased, and the central and southern regions of the country report most occurrences (*1*,*4*).

This study reports the emergence of urban transmission of feline sporotrichosis in Puerto Iguazú, Misiones, Argentina (25°36′39″S, 54°34′49″W). Located in the extreme northeast of Argentina, on the border of the Triple Frontier, the city has a population of ≈54,675 people and is 1,278 km from Buenos Aires. Puerto Iguazú is a major tourist destination because of the Iguazú Falls and is characterized by substantial cross-border dynamics, including high population and commercial movement.

During August 2023–February 2024, we conducted an intensified passive surveillance on 21 domestic cats (*F. catus*) from 12 households in the urban area of Puerto Iguazú (Figure). We included cats with lesions consistent with feline sporotrichosis (ulcers, scabs, soft nodules, and ulcerated subcutaneous nodules with exudate) and other cats without lesions but in contact with affected cats or living in areas with documented cases. No cats received treatment before sample collection. Pet owners provided written consent, and we recorded clinical, epidemiologic, and demographic data. Veterinarians evaluated all suspected cases and unaffected cats in contact with affected cats, and ongoing prevention and awareness campaigns provided information to owners and the community.

**Figure:**
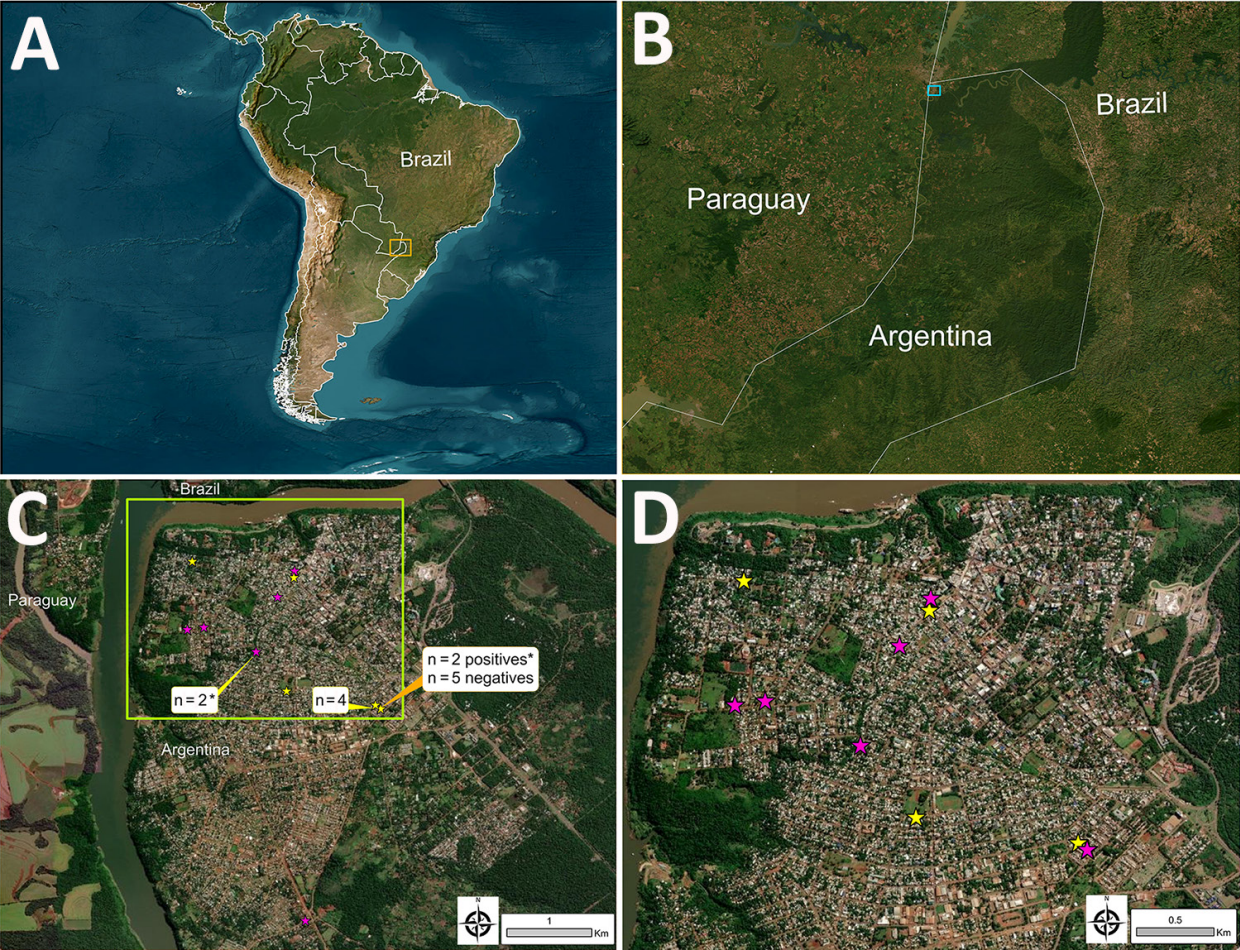
Spatial distribution of feline sporotrichosis near Brazil border, Argentina, 2023–2024. A, B) Relative position in South America showing the location of the Triple Frontier (A) and province of Misiones, Argentina (B). C) Puerto Iguazú within Argentina, showing positive confirmed feline sporotrichosis cases (n = 9; pink stars) and ruled-out cases (n = 12; yellow stars). D) Enlarged image of yellow box in panel C. For those areas where multiple cases (positive and negative) overlap in location, the number of individual cases is indicated. *Cases where intrahousehold transmission occurred between 2 cats (FSCMi24–FSCMi25 and FSCMi41–FSCMi43). In the spatial analysis, the distance between households with feline sporotrichosis cases was measured by using nearest-neighbor calculation in ArcGIS Pro (https://pro.arcgis.com), which calculates the shortest distance between 2 points.

We collected nasal and lesion swab specimens from cats with skin lesions and nasal swabs from cats without lesions. We conducted diagnosis and species identification of *Sporothrix* by using phenotypic (Giemsa stain and culture) and genotypic methods (sequencing internal transcribed spacer region and partial sequencing of the calmodulin gene) (*1*,*4*,*10*).

We studied 21 cases. Nine (42.9%) were suspected cases, and 12 (57.1%) were unaffected cats. We confirmed feline sporotrichosis by culture in 77.8% (7/9) of the suspected cases and 16.7% (2/12) of the unaffected cats (Appendix Figure). We confirmed *S. brasiliensis* in 77.8% (7/9) cases by using molecular analysis. Two samples were inconclusive because of mold and bacterial contamination, which hindered identification of *Sporothrix* species (Table). Among the 2 confirmed cases in unaffected cats, we identified sneezing as the sole symptom in 1 case, and we ruled out 2 suspected cases because of differential diagnosis (dog bite and dermatophytosis) (Table). We reported all cases to health authorities.

**Table:** Features of domestic cats (*Felis catus*) suspected of feline sporotrichosis near Brazil Border, Argentina, 2023–2024*

HH no.	Cat ID	Date	Age/sex	Altered	Habitation†	Type, location of lesion	Collected material	DE, culture	Species, strain no.
1	FSCMi-011‡	2023 Aug 1	Adult/M	No	Feral	Multiple dog bites	NS, LS, C	N/N	NA
2	FSCMi-017	2023 Nov 13	Adult/M	Yes	Outdoor	NI	NS, C	N/N	NA
	FSCMi-024	2023 Nov 24	Adult/M	Yes	Outdoor	NI	NS	ND, *Sporothrix* sp.	*S. brasiliensis*, 247479
	FSCMi-025§	2023 Nov 13	Adult/M	Yes	Outdoor	NI	NS	N, *Sporothrix* sp.	*S. brasiliensis*, 247478
	FSCMi-019	2023 Nov 13	Adult/F	Yes	Outdoor	NI	NS	N/N	
3	FSCMi-021	2023 Nov 16	Adult/F	No	Indoor	Localized, head	NS, LS	Yeast, *Sporothrix* sp.	NC
4	FSCMi-023	2023 Nov 24	Adult/M	Yes	Outdoor	Multiple, head, dorsum, and extremities	NS, LS	ND, *Sporothrix sp*.	*S. brasiliensis*, 247481
5	FSCMi-026	2023 Nov 24	Adult/M	Yes	Feral	Localized, head	NS, LS	ND, *Sporothrix* sp.	*S. brasiliensis,* 247480
6	FSCMi-031	2023 Dec 20	Kitten/M	No	Outdoor	NI	NS	N/N	NA
	FSCMi-032	2023 Dec 20	Juvenile/?	No	Outdoor	NI	NS	N/N	NA
	FSCMi-033	2023 Dec 20	Kitten/M	No	Outdoor	NI	NS	N/N	NA
7	FSCMi-034	2023 Dec 20	Adult/M	Yes	Outdoor	NI	NS	N/N	NA
	FSCMi-035	2023 Dec 20	Adult/M	Yes	Outdoor	NI	NS	N/N	NA
	FSCMi-036	2023 Dec 20	Adult/M	Yes	Outdoor	NI	NS	N/N	NA
	FSCMi-037	2023 Dec 20	Adult/F	Yes	Outdoor	NI	NS	N/N	NA
8	FSCMi-038	2023 Dec 20	Juvenile/F	Yes	Outdoor	NI	NS	N/N	NA
9	FSCMi-040	2024 Jan 10	Juvenile/F	No	Feral	Multiple, head and extremities	NS, LS	Yeast, *Sporothrix* sp.	NC
10	FSCMi-041	2024 Jan 15	Adult/M	Yes	Outdoor	Multiple, head, dorsum, and extremities	NS, LS	Yeast, *Sporothrix* sp.	*S. brasiliensis*, 247599
	FSCMi-043	2024 Jan 22	Adult/M	Yes	Outdoor	Multiple, head and extremities	NS, LS	Yeast, *Sporothrix* sp.	*S. brasiliensis*, 247600
11	FSCMi-042‡	2024 Jan 15	Juvenile/F	No	Outdoor	Alopecia	NS, LS	ND, *Microsporum canis*	NA
12	FSCMi-049	2024 Feb 28	Senior/M	Yes	Outdoor	Multiple, head, dorsum, and extremities	NS, LS	Yeast, *Sporothrix* sp.	*S. brasiliensis*, 247735

*C, claw; DE, direct examination of the sample with Giemsa staining; HH, household; LS, lesion swab; N, negative; NA, not applicable; NC, inconclusive because of mold and bacterial contamination; ND, not determined; NI, no injuries; NS, nasal swab specimen; ?, unknown. †Habitation: indoor, cats kept exclusively indoors or in enclosed spaces without outdoor access; outdoor, cats with free access to roam outdoors, including streets, neighboring properties, or vacant lots; feral, cats living in the wild with minimal or no human interaction. ‡Dog bites and diagnosis of dermatophytosis. §The cat had frequent sneezing.

We identified feline sporotrichosis cases in 58.3% (7/12) of the households. In 2 households, we detected multiple cases, suggesting intradomestic transmission. However, in 5 households, we found only 1 cat with sporotrichosis. Most of the cats had free access to streets, neighboring properties, and vacant lots. The average distance between the nearest households with feline sporotrichosis cases was 1.84 ± 1.22 km (range 0–4.54 km) (Table; Figure).

Our results describe a large outbreak of *S. brasiliensis* in Argentina. We identified 9 proven cases of feline sporotrichosis in 7 months in Puerto Iguazú, in the province of Misiones, which is more than reported for the whole country (*1*). The outbreak reflects transmission dynamics similar to the epidemic in Brazil. Evidence shows multiple foci of transmission and asymptomatic carriers spreading the *S. brasiliensis* fungus (*2*,*5*,*7*,*10*). In addition, the nearest epidemic focus is on the Brazil side of the Triple Frontier (*7*), and to our knowledge, Buenos Aires and Santa Cruz reported the latest cases of feline sporotrichosis in Argentina (*1*).

Asymptomatic carriers hinder sporotrichosis control efforts by delaying diagnosis and treatment. Screening all contacts of confirmed cases is essential to minimize the risk for transmission (*1*,*10*). Addressing those challenges requires mandatory case reporting and public health measures. The detection and control of the expansion of feline sporotrichosis outside Brazil in contiguous countries requires coordinated cross-border One Health actions and context-specific interventions, which will be crucial to safeguard local communities and tourists (*1*,*5*).

Appendix Feline sporotrichosis and phenotypic studies for spatial distribution of feline sporotrichosis near Brazil Border, Argentina, 2023–2024.
