# Supplementary material for: Emergence of Feline Sporotrichosis near Brazil Border, Argentina, 2023–2024
Source: Emerg Infect Dis. 2025 May;31(5):1045–8. doi: 10.3201/eid3105.241882 (PMC12044224; doi:10.3201/eid3105.241882)
Supplement: Appendix — Feline sporotrichosis and phenotypic studies for spatial distribution of feline sporotrichosis near Brazil Border, Argentina, 2023–2024. [file 24-1882-Techapp-s1.pdf]

Article DOI: https://doi.org/10.3201/eid3105.241882

EID cannot ensure accessibility for supplementary materials supplied by authors. Readers who have difficulty accessing supplementary content should contact the authors for assistance.

Emergence of Feline Sporotrichosis near Brazil Border, Argentina, 2023–2024

Appendix

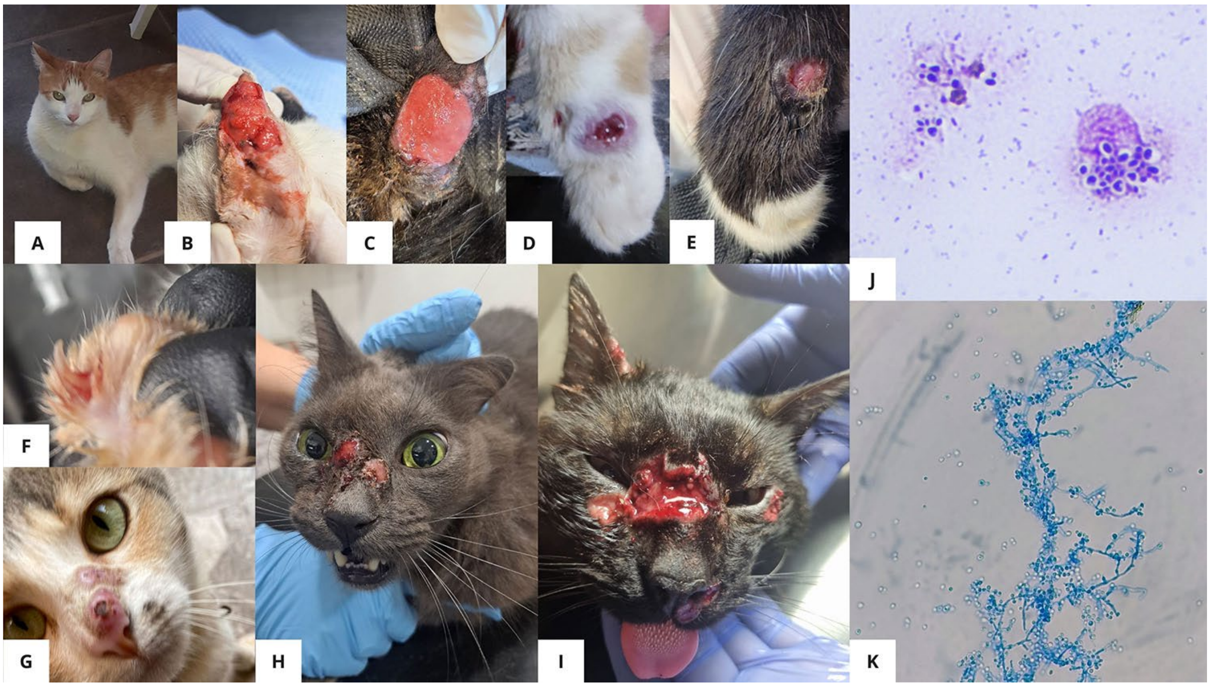

Appendix Figure. Feline sporotrichosis and phenotypic studies. A) Cat without lesions, with frequent sneezing (FSCMi25). B, C) Multiple ulcerative lesions in the ear (FSCMi021 and FSCMi026). D, E) Ulcerative lesion on forelimbs (FSCMi043 and FSCMi049). F) Ulcerative lesion around the claw (FSCMi040). G) Crusty ulcerative lesions on the nose (FSCMi043). H, I) Multiple crusty ulcerative lesions on the head (FSCMi041 and FSCMi026). J) Cigar-shaped oval capsulated yeasts (Giemsa stain; magnification ×1,000). K) Micromorphology of *Sporothrix* sp. colonies (lactophenol cotton blue; magnification ×400).
